# Reasons For Not Performing Keratorefractive Surgery in Patients Seeking Refractive Surgery in a Hospital-Based Cohort in “Yemen”

**DOI:** 10.4103/0974-9233.71605

**Published:** 2010

**Authors:** Mahfouth A. Bamashmus, Mahmoud F. Saleh, Mohamed A. Awadalla

**Affiliations:** 1 Eye Department, Faculty of Medicine and Health Sciences, Sana’a University, Egypt; 2 Department of Cornea and Refractive Unit, Magrabi Eye Hospital, Sana’a, Republic of Yemen, Egypt; 3 Eye Department, Faculty of Medicine, Al-Azhar University, Egypt

**Keywords:** Keratorefractive Surgery, Laser *In Situ* Keratomileusis, Myopia, Photorefractive Keratectomy, Yemen

## Abstract

**Background:**

To determine and analyze the reasons why keratorefractive surgery, laser *in situ* keratomileusis (LASIK) and photorefractive keratectomy (PRK) were not performed in patients who presented for refractive surgery consultation.

**Materials and Methods:**

A retrospective observational study was performed between January 2006 and December 2007 in the Yemen Magrabi Hospital. The case records of 2,091 consecutive new patients who presented for refractive surgery were reviewed. Information from the pre-operative ophthalmic examination, such as refractive error, corneal topography and visual acuity, were analyzed. The reasons for not performing LASIK and PRK in the cases that were rejected were recorded and analyzed.

**Results:**

In this cohort, 1,660 (79.4%) patients were advised to have LASIK or PRK from the 2,091 patients examined. LASIK and PRK were not advised in 431 (21%) patients. The most common reasons for not performing the surgery were high myopia >-11.00 Diopters (19%), keratoconus (18%), suboptimal central corneal thickness (15%), cataract (12%) and keratoconus suspect (forme fruste keratoconus) (10%).

**Conclusion:**

Patients who requested keratorefractive surgery have a variety of problems and warrant comprehensive attention to selection criteria on the part of the surgeon. Corneal topographies and pachymetry of refractive surgery candidates need to be read cautiously. High-refractive error, keratoconus and insufficient corneal thickness were found to be the leading reasons for not performing keratorefractive surgery in this study.

## INTRODUCTION

Laser *in situ* keratomileusis (LASIK) and photorefractive keratectomy (PRK) have gained popularity as the surgical procedures of choice for the correction of myopia.^1^ The safety and efficacy of LASIK and PRK are well established.^2^,^5^

Meticulous pre-operative screening of potential candidates is a key factor contributing to successful outcomes in refractive surgery. A candidate for refractive surgery must undergo a comprehensive ophthalmic examination. Each step of the pre-operative evaluation is fundamental to the decision to perform or avoid a keratorefractive procedure. A number of patients are excluded from corneal laser refractive surgery due to corneal disease, thin corneas or a variety of other reasons.^6^ Patients who are not candidates for refractive surgery are often offered safer alternatives such as phakic intraocular lens implantation and clear lens extraction if corneal topography is mildly assymetric or normal.^6^,^7^

In this study, we retrospectively analyzed the reasons why LASIK and PRK surgeries were not performed in patients seeking refractive surgery in Yemen.

## MATERIALS AND METHODS

This is a retrospective observational study. The clinical and investigational findings were reviewed and the reasons for not performing keratorefractive surgery (LASIK or PRK) were recorded and analyzed. The study was approved by the Research and Ethics Committee of Yemen Magrabi Hospital and the procedures followed were in accordance with the ethical standards of the responsible committee on human experimentation (institutional or regional) and with the Declaration of Helsinki 1975, as revised in 2000.

The medical records of 2,091 consecutive patients who presented for a refractive consultation at the refractive surgery unit in Yemen Magrabi Hospital, Sana’a, between January 2006 and December 2007 were reviewed. Potential candidates were provided an educational booklet followed by a consultation with the surgeon to discuss any further questions or concerns. All patients underwent routine screening protocols. Pre-operative data evaluated in this study were uncorrected visual acuity (UCVA), best spectacle-corrected visual acuity (BSCVA), refractive error (subjective and cycloplegic), mesopic pupil size, slitlamp biomicroscopy, dilated retinal exam, ultrasonic pachymetry (Nidek UP-1000 Ultrasonic Pachymeter, Nidek Co. Ltd., Gamagori, Japan), keratometry and corneal topography (TMS-2, Tomey Co. Ltd., Nagoya, Japan).

The selection criteria for LASIK or PRK in the refractive surgery unit are presented in Table 1. LASIK or PRK was not performed in patients who do not meet these criteria.

**Table 1:** Parameters used for patient selection for keratorefractive surgery

Age 18 years or older Patients approaching presbyopic age are informed about the requirement of wearing spectacles for near correction Stable refraction for the previous year or longer Absence of any corneal pathology (keratoconus index [KCI] of the TMS-2 topography system was used for keratoconus screening) - Keratoconus - Keratoconus suspect (forme-fruste keratoconus) - Pellucid marginal degeneration Absence of any other ocular pathology - Herpes keratitis - Corneal dystrophy or degeneration - Lens opacities - Retinal pathology - Glaucoma suspect - Dry eyes Absence of medical contraindications - Diabetes mellitus - Autoimmune disease - Immunocompromised status No pregnancy or lactation LASIK - Myopia of up to -11.00 Diopters (D) - Astigmatism <4.00 Diopters (D) - Hyperopia <4.00 Diopters (D) - Central corneal thickness (CCT) of >480 μm - Presumed residual stromal bed thickness (RSB) of >250 μm PRK - Myopia of up to -5.00 Diopters (D) - Astigmatism <2.00 Diopters (D) - CCT of >450 μm - Remaining corneal thickness >400 µm Written consent after detailed discussion with the patient

Corneal topography was performed prior to measuring central corneal thickness (CCT). The Klyce-Maeda keratoconus index (KCI) from the TMS-2 was used to detect early keratoconus.^8^,^9^ Three topographic measurements of each eye were performed and the technician chose the image with the widest corneal coverage for processing. If artifacts were present on the raw images (mire reflection) or on the color-coded topographic maps, the measurement was repeated.

If the KCI was higher than 5%, LASIK and PRK were not performed. Patients with topographic signs of keratoconus, forme-fruste keratoconus or pellucid marginal degeneration in one eye were denied surgery. In cases where the KCI was 0% yet the corneal topography was asymmetric and the CCT was normal, the patients were offered PRK only.

Ultrasonic pachymetry (Nidek US 1000 pachymeter, Nidek Co.) was used to measure the CCT=of all eyes. Three serial measurements were performed and the lowest reading was recorded. Regional pachymetry (superior, inferior, nasal and temporal measurements) were performed in suspect cases or cases with asymmetric topographies.

If the pre-operative CCT was >480 *μ*m and the residual stromal bed thickness was higher than 250 *μ*m, the patient was considered a candidate for LASIK. In cases were the pre-operative CCT was lower than 480 *μ*m or the residual stromal bed thickness was lower than 250 *μ*m, the patient was advised to undergo PRK and not LASIK [Table 2]. In cases were the corneal thickness was <450 *μ*m or the refractive error precluded safe residual corneal thickness, phakic intraocular lens implantation or clear lens extraction was recommended if the corneal topography was normal or had very mild asymmetry.

**Table 2:** Comparison of patient profiles that presented for refractive surgery

		Operated by LASIK or PRK	Not operated	Validation
Sex	Male	787	184	OR = 1.19 (95% CI,
	Female	873	247	0.96-1.47)
Age	Mean	26.85	27.93	1.08 (0.24-1.92)
	SD	5.87	8.35	
Right eye myopia	Mean	3.23	7.04	3.81 (3.45-4.16)
	SD	1.82	6.35	
Left eye myopia	Mean	3.2	6.59	3.39 (2.81-3.96)
	SD	1.81	6.00	
Right eye corneal thickness	Mean	528.28	503.94	25.28 (22.3-28.2)
	SD	25.66	32.72	
Left eye corneal thickness	Mean	529.45	504.78	24.67 (21.8-28.0)
	SD	25.84	32.23	
Right eye anterior vertical corneal curvature	Mean	44.67	46.82	2.15 (1.99-2.31)
	SD	1.42	1.94	
Right eye anterior horizontal corneal curvature	Mean	43.60	44.22	0.62 (0.45-0.79)
	SD	1.41	2.21	
Left anterior vertical corneal curvature	Mean	44.70	45.85	1.15 (0.96-1.34)
	SD	1.45	2.78	
			
Left anterior horizontal corneal curvature	Mean	43.62	44.22	0.93 (0.76-1.10)
	SD	1.41	2.17	

SD = standard deviation

One or two drops of tropicamide 1% (Mydriacyl, Alcon laboratories Inc., USA) were instilled for mydriasis and cycloplegia. Cataract was detected by slitlamp examination after pupil dilation and any lens opacity was a contraindication for keratorefractive surgery. Other ocular abnormalities and systemic diseases considered contraindications for keratorefractive surgery are listed in Table 2.

## RESULTS

The study cohort comprised 971 (46%) males and 1,120 (54%) females, with a mean age of 26 ± 5.87 years (range, 18–54 years). Among the 2,091 patients who requested refractive surgery, 1,660 (79%) met the inclusion criteria for LASIK or PRK. Of those who initially requested surgery, 431 (21%) were poor candidates for LASIK or PRK and did not undergo surgery.

Table 2 compares the profile of patients who presented for refractive surgery. The most common reasons for not performing keratorefractive surgery were high myopia >-11.00 Diopters (19.5%), keratoconus (17.9%), suboptimal CCT (14.6%), cataract (12.5%) and keratoconus suspect (forme-fruste keratoconus) (10.4%) [Table 3].

A total of 18 (4%) patients were excluded due to hyperopia >4 Diopters. Four cases were excluded due to high astigmatism (>4 Diopters), all with suspicious corneal topography (forme-fruste keratoconus) and were included in the forme-fruste category in Table 3.

**Table 3:** Reasons for not performing keratorefractive surgery (laser *in situ* keratomileusis or photorefractive keratectomy)

Reason for rejection	Number (%)
High myopia > -11.00 Diopters	84 (19.5)
Keratoconus	77 (17.9)
Suboptimal corneal thickness	63 (14.6)
Cataract	54 (12.5)
Keratoconus suspect (FFK)	45 (10.4)
Myopia + suspicious topography	26 (6.0)
Suboptimal corneal thickness + suspicious	20 (4.6)
topography	
High hyperopia (>4 D)	18 (4.2)
Small prescription	11 (2.6)
Young age <18 years	10 (2.3)
Pellucid marginal degeneration	7 (1.6)
Retinitis pigmentosa	5 (1.2)
Presbyopia	5 (1.2)
Stargardt’s disease	2 (0.5)
Glaucoma	2 (0.5)
Old retinal detachment	1 (0.2)
Pregnancy	1 (0.2)
Total	431 (100)

FFK = Forme-fruste keratoconus

## DISCUSSION

LASIK and PRK with the excimer laser are now the two most common keratorefractive procedures performed for correction of myopia and myopic astigmatism.^10^,^11^ The pre-operative investigations associated with patient’s expectations and needs are key factors in the success of keratorefractive surgery. Identification of high-risk cases using corneal topography and pachymetry is an important component in this process. Corneal topography is the most sensitive method for the detection of ectatic corneal disorders.^9^,^12^

The majority of patients who had keratorefractive surgery recommend the procedure to their relatives and friends. However, the post-operative pain associated with PRK has resulted in most patients recommending LASIK.^13^,^14^ LASIK continues to be the preferred procedure by the majority of refractive surgeons.^10^,^11^ However, a sizeable minority of surgeons continue to prefer PRK.^4^ LASIK appears to have better efficacy and safety compared with PRK. However, the equality of outcomes remains contentious.^15^ Both procedures have gained popularity, but there are limitations of LASIK and PRK surgery. Refractive surgeons should adhere to the guidelines and avoid performing cases with contraindications of these procedures.^6^,^16^,^17^

One-fifth of the patients (21%) who presented at our refractive surgery unit were advised not to undergo keratorefractive surgery. Our search of the literature found only one report by Hori-Komai *et al*.^18^ in Japan that discussed the proportion of patients who request keratorefractive surgery but did not undergo surgery. High myopia, suboptimal corneal thickness and keratoconus accounted for 20.7%, 8.2% and 6.4% of the excluded cases, respectively. In our study, the proportion of keratoconus and suboptimal corneal thickness were higher than that in the Hori-Komai *et al*. study.^18^ This difference is likely due to the different populations in our study (Yemeni), where keratoconus is more common compared with Hori-Komai *et al*.’s study in the Japanese. Only the corneal characteristics that resulted in the exclusion of refractive surgery candidates were discussed by Ambrosio *et al*.^19^ However, comparison with our study is difficult as different criteria were used by Ambrosio *et al*.

The upper limit for performing LASIK in our hospital is -11.00 Diopters of myopia and 4.00 Diopters of astigmatism. Patients exceeding this limit are advised to have phakic intraocular lens implantation or clear lens extraction. Candidates with greater than +4.00 Diopters of hyperopia were also rejected from having keratorefractive surgery because of unstable post-operative refraction (regression), excessive central corneal steepening and higher rate of corneal haze after PRK. Some patients with refractive errors less than -11.00 Diopters and insufficient corneal thickness for complete correction were also advised to have phakic intraocular lenses.

Topographic analysis frequently yields characteristic clues to the presence of suspicious or ectatic diseases prior to the development of signs. Pre-operative topographical screening is considered the standard of care for all refractive surgical procedures.^12^,^19^ If LASIK or PRK is performed in corneas with topographic abnormalities, loss of vision may result due to ectasia.^7^,^20^

These patients are dissatisfied with the quality of vision even with glasses or contact lenses, providing even greater impetus to seek treatment at refractive surgery clinics. Keratoconus, keratoconus suspect and pellucid marginal degeneration are considered high-risk corneal diseases for the development of progressive keratectasia and have poor visual outcomes.^6^,^21^ If the keratoconus index (KCI) in the TMS-2 was >5%, LASIK and PRK were contraindicated and the patient was offered alternative procedures. Hence, all patients who had abnormality in the keratoconus screening program of the TMS-2, even if it was minimal, were rejected from keratorefractive surgery.^9^,^12^

In this series, high myopia (greater than -11.0 Diopters) was the most common reason (20%) for not offering patients’ keratorefractive surgery. Keratoconus was the second most common reason and accounted for 18%. If we sum all corneal topography abnormalities listed in Table 3, i.e. keratoconus, keratoconus suspect, pellucid marginal degeneration and suspicious topography with other reasons for rejection, they all together accounted for 40% of the cases that were poor candidates for keratorefractive surgery. An investigation by Varssano and coworkers to estimate the extent of candidate rejection based on topography alone reported that 70 eyes of the 200 studied eyes (35%) were rejected based on abnormality on corneal topography maps^12^ similar to our results.

We use the minimum CCT of 480 *μ*m for cases undergoing LASIK and >450 *μ*m in cases of PRK Table 2. Pachymetry is an important test and is an indicator of corneal health, but varies widely in different ethnic populations.^22^ Patients with thin corneas, normal corneal topography and refraction less than -5.00 Diopters of myopia or thick cornea with mild atypical corneal topographies were advised to have PRK.^23^ Patients with thin corneas and higher refractive errors and cases with >5% Klyce-Maeda KCI using the TMS-2, regardless of their refractive error, were advised to have phakic intraocular lens implantation.^24^ Abnormally thin corneas (<450 *μ*m) were excluded from having keratorefractive surgery.

Safe CCT and safe residual stromal bed thickness is of special importance because Yemeni patients generally have thinner corneas (mean of 521 *μ*m).^25^ It is essential to measure pre-operative CCT and to measure intraoperative pachymetry after flap reflection during LASIK as the actual flap thickness of the flap may be significantly different from the intended thickness due to variability of the microkeratome. This also allows the surgeon to determine the standard deviation of the microkeratome in order to plan the appropriate flap thickness. To date, the lower limit for a stable bed thickness remains uncertain. Yet, the majority of refractive surgeons consider 250 *μ*m as the limit.^26^ However, iatrogenic keratectasia has been reported in cases with residual stromal bed thickness of >275 *μ*m.^27^ Barraquer *et al*. suggested that a residual corneal thickness of 300 *μ*m should remain to prevent ectasia.^28^ The residual stromal bed depth is dependent on pre-operative CCT, the thickness of the flap and the depth of laser ablation.^29^

The most serious long-term complication of keratorefractive surgery is the weakening of the cornea and the development of keratectasia after LASIK and PRK.^7^,^30^,^31^ If excessive corneal tissue is removed during LASIK or the cornea is too thin for the desired ablation, the biomechanically compromised cornea is at risk of developing iatrogenic keratectasia.^30^,^32^ This is also true if LASIK is performed in keratoconus suspects or high myopes requiring a large volume of tissue removal resulting in residual stromal bed thickness of <250 *μ*m.^33^ The risk of ectasia after PRK is much lower than LASIK,^34^ but there are our associated complications with PRK, such as a higher risk of infectious keratitis and corneal haze in eyes treated for higher refractive errors.^4^,^35^

Pre-presbyopes and presbyopes were informed of the requirement of glasses for near work post-operatively. In this subpopulation, some agreed to undergo LASIK while others considered the surgery pointless. We do not advocate monovision treatment to our patients and advise them to wait for newer technology such as PresbyLASIK or other promising laser algorithms.^36^

Careful screening reduces suboptimal results and mitigates frustration for the refractive surgeon and patient. Refractive surgeons should be meticulous in evaluating corneal topography to detect features consistent with ectasia or other suspect cases. If the possibility of an unsatisfactory outcome exists due to some of the parameters found during pre-screen, refractive surgeons should advise their patients not to have the procedure. Even with careful consideration of the indications and contraindications of the LASIK and PRK, some patients can develop complications.

In general, the majority of our patients had satisfactory outcomes and have been very satisfied. For those patients who are not suitable for keratorefractive surgery, other alternative refractive procedures such as phakic intraocular lens implantation and clear lens extraction should be presented.
